# Bacteria-phage coevolution with a seed bank

**DOI:** 10.1038/s41396-023-01449-2

**Published:** 2023-06-07

**Authors:** Daniel A. Schwartz, William R. Shoemaker, Andreea Măgălie, Joshua S. Weitz, Jay T. Lennon

**Affiliations:** 1 Department of Biology, Indiana University, Bloomington, Indiana, IN USA; 2 The Abdus Salam International Centre for Theoretical Physics (ICTP), Trieste, Italy; 3 School of Biological Sciences, Georgia Institute of Technology, Atlanta, GA USA; 4 Interdisciplinary Graduate Program in Quantitative Biosciences, Georgia Institute of Technology, Atlanta, GA USA; 5 School of Physics, Georgia Institute of Technology, Atlanta, GA USA; 6 Institut de Biologie, École Normale Supérieure, Paris, France

**Keywords:** Microbial ecology, Molecular evolution

## Abstract

Dormancy is an adaptation to living in fluctuating environments. It allows individuals to enter a reversible state of reduced metabolic activity when challenged by unfavorable conditions. Dormancy can also influence species interactions by providing organisms with a refuge from predators and parasites. Here we test the hypothesis that, by generating a seed bank of protected individuals, dormancy can modify the patterns and processes of antagonistic coevolution. We conducted a factorially designed experiment where we passaged a bacterial host (*Bacillus subtilis*) and its phage (SPO1) in the presence versus absence of a seed bank consisting of dormant endospores. Owing in part to the inability of phages to attach to spores, seed banks stabilized population dynamics and resulted in minimum host densities that were 30-fold higher compared to bacteria that were unable to engage in dormancy. By supplying a refuge to phage-sensitive strains, we show that seed banks retained phenotypic diversity that was otherwise lost to selection. Dormancy also stored genetic diversity. After characterizing allelic variation with pooled population sequencing, we found that seed banks retained twice as many host genes with mutations, whether phages were present or not. Based on mutational trajectories over the course of the experiment, we demonstrate that seed banks can dampen bacteria-phage coevolution. Not only does dormancy create structure and memory that buffers populations against environmental fluctuations, it also modifies species interactions in ways that can feed back onto the eco-evolutionary dynamics of microbial communities.

## Introduction

Coevolution arises from reciprocal evolutionary changes between two or more species. Common among mutualists, coevolution is also an important process for understanding host-parasite dynamics [1]. For example, antagonistic interactions tend to involve selection for defense strategies, whether they be behavioral attributes or morphological characteristics, that diminish the negative effects of a parasite on host fitness [2–4]. In turn, parasites often evolve to overcome a host’s investment into defensive strategies [5, 6]. These underlying mechanisms of coevolution can give rise to eco-evolutionary feedbacks that have implications for the diversity and dynamics of coupled populations [7–10]. The complexity of coevolution is further influenced by demographics [11], mating systems [12], nutrition [13], productivity [14], dispersal [15], and other traits that contribute to organismal fitness [16].

One trait that may affect coevolution is dormancy. When challenged by fluctuating or suboptimal conditions, many organisms interpret environmental cues and responsively transition into a metabolically inactive state. Organisms can also hedge their bets in unpredictably noisy environments by stochastically transitioning between metabolic states [17, 18]. With either strategy, dormancy creates a reservoir of inactive individuals known as a “seed bank”. Although not capable of reproducing, dormant individuals enjoy reduced rates of mortality. As a result, seed banks affect the evolution and ecology of populations. For example, genetic drift is reduced with a seed bank because it increases the effective population size [19–21]. In addition, seed banks retain individuals in a population that would otherwise be vulnerable to removal by natural selection [22, 23]. Taken together, the genetic storage provided by seed banks can buffer lineages from extinction and contribute to the maintenance of diversity within a population [24].

Seed banks also alter species interactions in ways that may affect coevolution. It is well established that dormancy allows competing species to coexist via the storage effect. This phenomenon reflects the ability of species to grow under favorable conditions while minimizing losses during unfavorable conditions owing to long-lived life stages [25, 26]. Similarily, seed banks modify the dynamics of antagonistically interacting species. On the one hand, dormancy may be reinforced by providing indirect benefits to predators and parasites. For example, the formation of resting structures by crustacean zooplankton (*Daphnia pulex*) can limit overgrazing of microbial resources and reduce the amplitude of predator-prey cycles [27]. On the other hand, observations from diverse taxa, ranging from bacteria to rodents, suggest that dormancy can provide prey with a refuge against organisms that infect or consume them [28–30]. Nevertheless, some parasites appear to have coopted host dormancy in a way that increases their survival and transmission [31–33]. While these observations have inspired theoretical work examining the interplay between dormancy and coevolution of host-parasite dynamics, empirical tests are lacking [34, 35].

For decades, communities of bacteria and phage have been used for testing coevolutionary theory [36]. Microbial strains can be assembled, replicated, and propagated in small volumes for hundreds to thousands of generations. Short generation times and large population sizes allow for rapid evolution, which can be tracked through longitudinal sampling [37]. In such studies, bacteria and phage often coexist owing in part to arms race dynamics and negative-frequency dependent selection, which has been demonstrated with infection networks and genome sequencing [38–40]. Growing interest in virus defense strategies provides an opportunity to gain new insight into bacteria-phage coevolution [41]. While many forms of virus defense, such as restriction modification and CRISPR-Cas, take place inside the cell [42, 43], other forms of resistance take place on the surface of the cell. In the latter case, targets of selection are often associated with the initial step of infection where tail like-structures of a phage particle attach to pilli, flagella, lipopolysaccharides, or other receptor molecules found on the cell’s outer membrane [38, 39, 44]. Ultimately, bacteria-phage coevolution is dependent on the genetic loci under selection and the fitness costs of evolved strains, along with physical or physiological refugia from phages [45–48].

Microbial systems also provide a means for testing how dormancy contributes to coevolution. One of the best understood forms of dormancy is endosporulation [49]. When challenged by resource limitation, bacteria like *Bacillus* and *Clostridium* undergo a complex development process that transforms an actively growing vegetative cell into a metabolically inert and long-lived dormant spore [50, 51]. The pathways controlling endosporulation are well characterized and amenable to genetic manipulation, which can be leveraged in experimental evolution trials [52, 53]. While endosporulation confers tolerance to a broad range of environmental stressors, it may also modify interactions with phages [54]. For example, the receptors needed for phage attachment are masked by the encasing spore coat [55], which could render bacteria resistant to infection. Nevertheless, viral parasites may be able to overcome this dormancy defense mechanism. Recent studies have demonstrated that some phages carry host-derived genes that can inhibit endosporulation and thus eliminate the potential refuge conferred by this type of phenotypic plasticity [56, 57].

In this study, we conducted experiments with a spore-forming bacterium (*Bacillus subtilis*) and its phage to test how dormancy and the resulting seed bank influences eco-evolutionary dynamics of a coevolving host and parasite. After engineering a mutation in an essential gene for sporulation, we tested how seed banks affect infection rates, population dynamics, and community stability. By isolating bacteria from different time points in the experiment, we tracked the maintenance of phage resistant phenotypes in the host population in the presence and absence of a seed bank. Using pooled population sequencing, we also quantified patterns of molecular diversity and genetic signatures of coevolution.

## Methods

### Strains and growth media

We used *Bacillus subtilils* 168 Δ6 (Table S1) as the bacterial host in our experiments. This engineered derivative of the model strain *B. subtilis* 168 has had all known prophages deleted from its genome and is capable of forming endospores [58]. From the Δ6 strain, we engineered a non-sporulating host by deleting *spoIIE*, a gene that is specific to, and essential for, endospore formation (see Supplementary Text). We confirmed that the *spoIIE* deletion did not affect fitness or alter phage infection (see Supplementary Text). We cultured the spore-forming (Δ6) and non-spore forming (Δ6 Δ*spoIIE*) bacteria in LB medium with low salt (5 g/L NaCl) or Difco sporulation medium (DSM [59]). Media were amended with chloramphenicol (5 µg/mL) to which the engineered *Bacillus* are resistant, agar (15 g/L) for plating, and CaCl_2_ (10 mM in LB and 1 mM in DSM) to facilitate phage adsorption. We used phage SPO1 as the parasite in our experiments (Table S1). This virulent phage belongs to the Herelleviridae family [60], a group of viruses with representatives that can infect *B. subtilis* and other Bacillota [61]. SPO1 has a dsDNA genome (132 kb) and myovirus-like morphology, including a long contractile tail and icosahedral head [62]. To amplify SPO1, we collected lysates from plate infections after flooding Petri dishes with pH 7.5 buffer (10 mM Tris, 10 mM MgSO_4_, 4 g/L NaCl, 1 mM CaCl_2_). We then cleared the phage-containing buffer from bacteria by centrifugation (7200 × *g*, 10 min) and filtration (0.2 μm).

### Phage adsorption assay

To characterize attachment to hosts, we conducted adsorption assays where we quantified the percentage of phage particles that attached to spores and vegetative cells over time [63]. We purified spores produced in an overnight culture in DSM by lysozyme treatment (50 µg/mL, 1 hr, 37 °C), followed by SDS treatment (0.05%) and three washes in H_2_O. To prevent germination, we resuspended purified spores in Tris-buffered saline (pH 7.5) lacking any resources required for germination. Vegetative cells were harvested from an overnight culture in LB medium, which were washed and resuspended in Tris-buffered saline (pH 7.5). To initiate an adsorption assay, we mixed 10^7^-10^8^ vegetative cells or purified spores with ~10^4^ phages (multiplicity of infection = 10^-3^-10^-4^) in a shaking incubator for 5 min at 37 °C before sampling. We immediately filtered samples (0.2 µm) to remove cells and adsorbed phages before measuring titer of unabsorbed phages by plaque assays. This involved double-layer plating with 0.3% agar overlays [64]. We measured the initial phage titer by setting up a control flask without cells. From phage abundances, we calculated the percent of adsorption, and tested whether these values were greater than zero using a one-sided *t*-test.

### Coevolution experiment

**Fig. 1:**
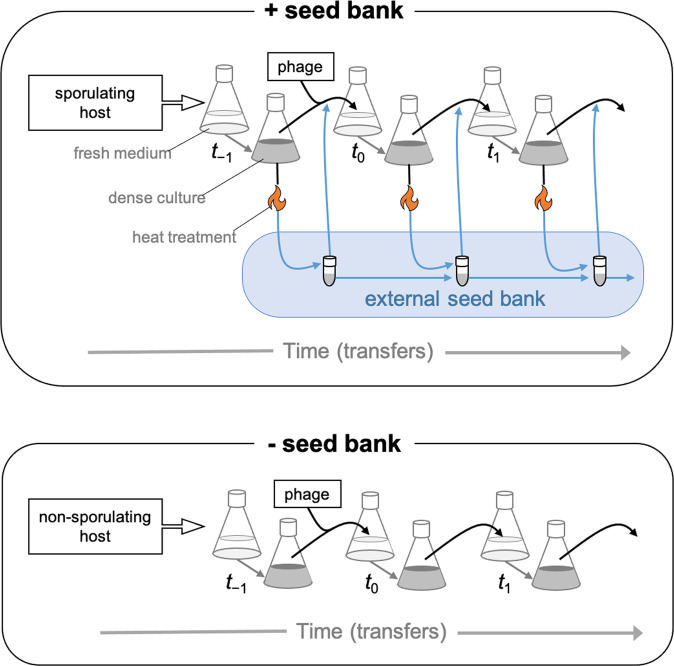
Illustration of seed bank manipulation. In the + *seed bank* treatment, we used a strain of *Bacillus subtilis* that was capable of forming endospores after resources were exhausted by growth (black arrows). In addition, we established an external seed bank (shown in blue) to extend endospore residence time. The first step of this process involved purifying endospores through heat treatment (flame = 80 °C, 20 min), which eliminated phages and vegetative cells from a sample taken from the focal population contained in a flask. Then, we mixed these endospores with endospores preserved from previous transfers that were obtained in the same fashion. This spore mixture (i.e., external seed bank) and an untreated sample taken from a focal population were used to inoculate fresh medium and establish the next transfer. In the - *seed bank* treatment, serial transfers (black arrows) were conducted with a mutant strain of *B. subtilis* that was not capable of producing endospores in rich medium after resource exhaustion owing to an engineered mutation in a gene that is essential for sporulation (*spoIIE*). After establishing the seed bank with an initial serial transfer (*t*_-1_), we began the experiment at *t*_0_ by infecting half of the populations with phage SPO1. For simplicity, non-infected controls are not shown. See methods for further details.

We conducted a 2 × 2 factorially designed experiment where we serially passaged bacteria and phage in the presence or absence of a seed bank (Fig. 1). For each experimental unit, we inoculated 10 mL of DSM with 100 μL of an overnight culture of *B. subtilis* that was started from a single colony of Δ6 (+ *seed bank* treatment) or non-spore-forming Δ6 Δ*spoIIE* (*- seed bank* treatment). Half of the experimental units (*n* = 3 for each seed bank treatment) were randomly assigned to an uninfected control group (*- phage*), while the others received 10^6^ plaque forming units (PFU) from an isogenic lysate of SPO1 to achieve a multiplicity of infection of 0.0002 (+ *phage*). Once treatments were established, we maintained all populations (*n* = 12) in 10 mL of DSM in 50 mL Erlenmeyer flasks in a shaking incubator (200 rpm) at 37 °C.

#### Preparation of an external seed bank

When grown in DSM, Δ6 rapidly depletes resources, which promotes sporulation. For example, endospores made up 65 ± 7% (mean ± SD, *n* = 3) of the population after 48 h of incubation. However, when transferred into fresh medium, these spores germinated at a rate of 50% h^-1^ (Fig. S1), which has the potential to limit the accumulation of dormant individuals from past transfers when populations are being serially transferred. Therefore, we generated an age-structured external seed bank, which allowed us to mix old and new endospores without loss to germination (Fig. 1). Endospores from the external seed bank could then be added back to the focal population in a way that extended the residence time of endospores in our experiment (see Supplementary Text). To start, we harvested and washed cells twice with equal volumes of phosphate buffered saline (pH = 7.4) in a centrifuge (8000 × *g*, 5 min) to remove residual medium which could trigger spore germination. Next, to isolate endospores, we heat-treated samples to kill phage and vegetative cells (80 °C, 20 min). At each transfer, isolated endospores were then added to the seed bank by mixing them with the seed bank endospores from the previous transfer at a volumetric ratio of 4:1 (new:old). Last, we added a sample of this newly mixed seed bank back to the focal population upon the next serial transfer (Fig. 1).

#### Serial transfer and sampling

In order to track population dynamics, we serially passaged bacteria and phage over time. Upon each transfer, we aliquoted 1% of the population (100 μL) to fresh medium in a new Erlenmeyer flask (Fig. 1). For populations assigned to the + *seed bank* treatment, we transferred 50 μL of an untreated population sample and 50 μL of the seed bank to control for total inoculum size. We transferred each population every other day for 28 days for a total of 14 transfers, which amounted to approximately 90 host generations. We sampled each experimental unit daily to quantify population sizes (see below). At each transfer (48 h), we preserved samples of the host and phage populations for assessment of phenotypic and molecular evolution (see below). Bacteria were preserved by adding glycerol (15% volume per volume) to a population sample prior to storage at −80 °C. For preservation of bacterial endospores, we stored external seed banks at 4 °C. For preservation of phage lysates, 5 mL of sample was cleared by centrifugation (7200 × *g*, 10 min) and the supernatant was stored at 4 °C with 0.1 mL chloroform.

### Population dynamics

We quantified bacterial densities with a flow cytometry assay that distinguished endospores from vegetative cells (non-spores) based on differential uptake of the nucleic acid stain SYBR green [65]. We quantified phage densities using a quantitative PCR (qPCR) assay with SPO1-specific primers (Table S2) alongside a standard curve made from a serial dilution of the ancestral phage lysate of known titer. With the resulting data, we tested for the main effects of phage treatment, seed bank treatment, and time, along with higher order interactions using repeated measures (RM)-ANOVA implemented with a linear mixed-effects model (R package nlme v3.1-149 [66]). To help meet assumptions, we transformed raw abundance data using the Box-Cox method (R package car v3.0-10 [67]). To account for lack of independence in repeated sampling of populations over time, we included an autoregressive moving-average correlation structure (corARMA(*p*,*q*)) with parameters selected based on Akaike Information Criterion (AIC). To identify differences among treatment combinations, we conducted a *post hoc* analysis based on estimated marginal means of the RM-ANOVA model using the *emmeans* R package (v1.5.1 [68]). See Supplementary Text for more details.

### Evolution of phage-resistance

To test how seed banks affected the evolution of phage-resistance, we characterized the susceptibility of bacteria from different time points to infection by the ancestral phage. Our assay involved spotting of a turbid bacterial culture with a pin replicator onto DSM plates containing a surface-spread of the ancestral SPO1 (Fig. S2). As a control, we spotted the same clones on DSM plates without phage. Bacterial clones that could grow on both plates were scored as resistant, while clones that grew only in the absence of phage were scored as susceptible. We challenged bacterial clones (*n* = 22 per population) isolated from samples preserved during the first four transfers of the coevolution experiment against the ancestral phage. Clones revived from the seed bank were tested in the same manner (*n* = 22 per population). We tested for the effects of seed bank treatment and clone origin (total population vs. seed bank) on the evolution of resistance to the ancestral phage using RM-ANOVA as described above.

### Molecular evolution of bacteria and phage with a seed bank

We performed pooled population sequencing to evaluate how the seed bank and phage treatments affected the molecular evolutionary dynamics of bacteria and phage populations. We extracted genomic DNA at the end of serial transfers 1, 4, 7, 10 and 14 of the coevolution experiment (see Supplementary Text). Paired-end libraries were constructed with a target minimal coverage of 100 with 2 × 38 bp reads for phage and 2 × 150 bp reads for the bacteria. Sequencing was performed using a NextSeq500 sequencer (Illumina). Mutations and their frequency were called using breseq [69] in polymorphism mode. To focus on mutations with the largest potential effect on fitness, we restricted our analyses to nonsynonymous mutations, insertions, and deletions. Mutation frequency trajectories over time were considered only for mutations that were detected in at least three time-points.

To compare the effect of phage and seed bank treatments on the genetic diversity of bacteria, we calculated the multiplicity (*m*) of each gene in each population [70]. In our implementation, multiplicity standardizes the number of mutations observed in a gene according to gene length and allows for comparisons across genes and populations. Given that few fixation events were observed, we weighed gene multiplicity by the median frequency of all mutations in that gene, excluding zeros. The multiplicity (*m*) of the *i*th gene in the *j*th population is then defined as $m_{i,j} = \frac{{\bar L}}{{L_i}}\mathop {\sum }\nolimits_{k \in i} f_{med\,j,k}$, where *L*_*i*_ is the number of nonsynonymous sites in the *i*th gene, $\bar L$ is the mean number of nonsynonymous sites among all genes, and *f*_*med j,k*_ is the median frequency with respect to time of mutation *k* in gene *i* in population *j*. To account for differences in the total number of mutations acquired across populations, we normalized *m* by the sum of *m* for all genes, $\tilde m_{i,j} = m_{i,j}/\mathop {\sum }\nolimits_i m_{i,j}.$ We compared the distributions of relative multiplicity across treatments using two-sample Kolmogorov-Smirnov tests with *p*-values obtained by permuting treatment labels. Last, we compared the composition of genes with mutations between treatments using Principal Coordinate Analysis (PCoA) with Bray-Curtis distance. We used PERMANOVA on the top five principal coordinates (explaining >90% variation) with the *adonis2* function in vegan v2.6-2 [71] with Euclidean distance and 10,000 permutations. Results from the PERMANOVA allowed us to test for the main effects of the seed bank and phage treatments along with their interaction on mutated gene composition.

To determine how coevolution was affected by a seed bank, we quantified the correlation between host and phage mutations trajectories over time [72]. We calculated Pearson’s correlation coefficients between pairs of mutation trajectories in corresponding host and phage populations. To minimize undue influence of zeros, trajectory pairs with less than three observations of non-zero frequencies in both host and phage populations were removed. To obtain null distributions (i.e., no coevolution), we randomly permuted time labels of observed trajectories before calculating correlation coefficients, as described above. All comparisons between distributions were performed using two-sample Kolmogorov-Smirnov tests with *p*-values obtained by permuting treatment labels. See Supplementary Text for more details.

## Results

### Dormancy provided a refuge from phage infection

**Fig. 2:**
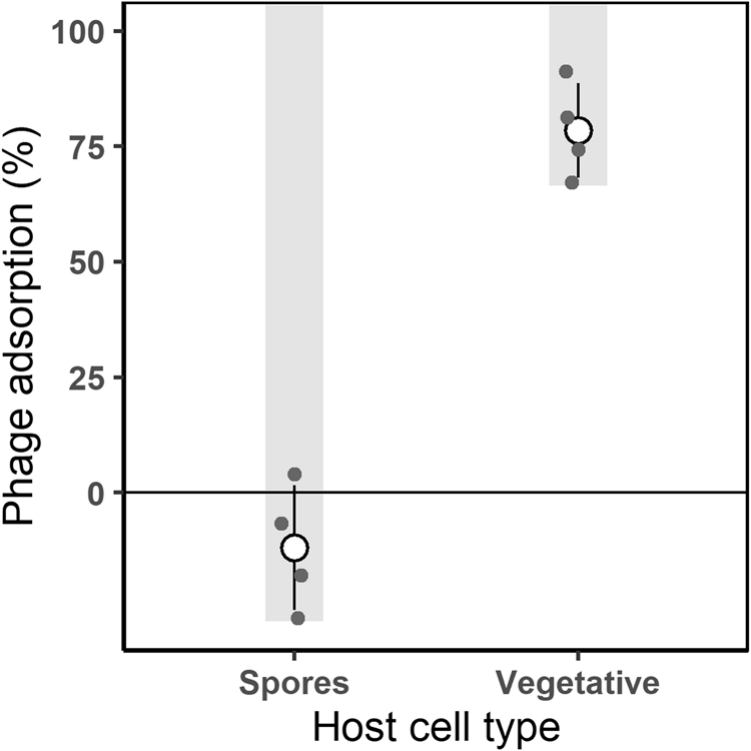
Endosporulation provided bacteria with a refuge from phages. We demonstrate that phage SPO1 cannot attach to endospores of wild type *Bacillus subtilis*. Percent adsorption was calculated from the decline in free phages over 5 min when mixed with either purified endospores or vegetative cells. Mean (○) and standard deviation of four biological replicates (●) are shown. Grey bars indicate the lower limit of the 95% confidence interval of a one-sided *t*-test for each of the host cell types.

Sporulation involves changes to the cell surface, which we predicted would reduce phage attachment. SPO1 phages were unable to adsorb to purified endospores (Fig. 2; one-sample *t*-test*, t*_3_ = −1.8, *p* = 0.91). In contrast, > 66% of SPO1 phages attached to vegetative cells produced by the same bacterial genotype within the first five minutes of the assay (one-sample *t*-test*, t*_3_ = 15.3, *p* = 0.0003) corresponding to an adsorption rate of 4.63 ( ± 3.07) × 10^−9^ mL^−1 ^min^−1^.

### Seed bank altered population dynamics

**Fig. 3:**
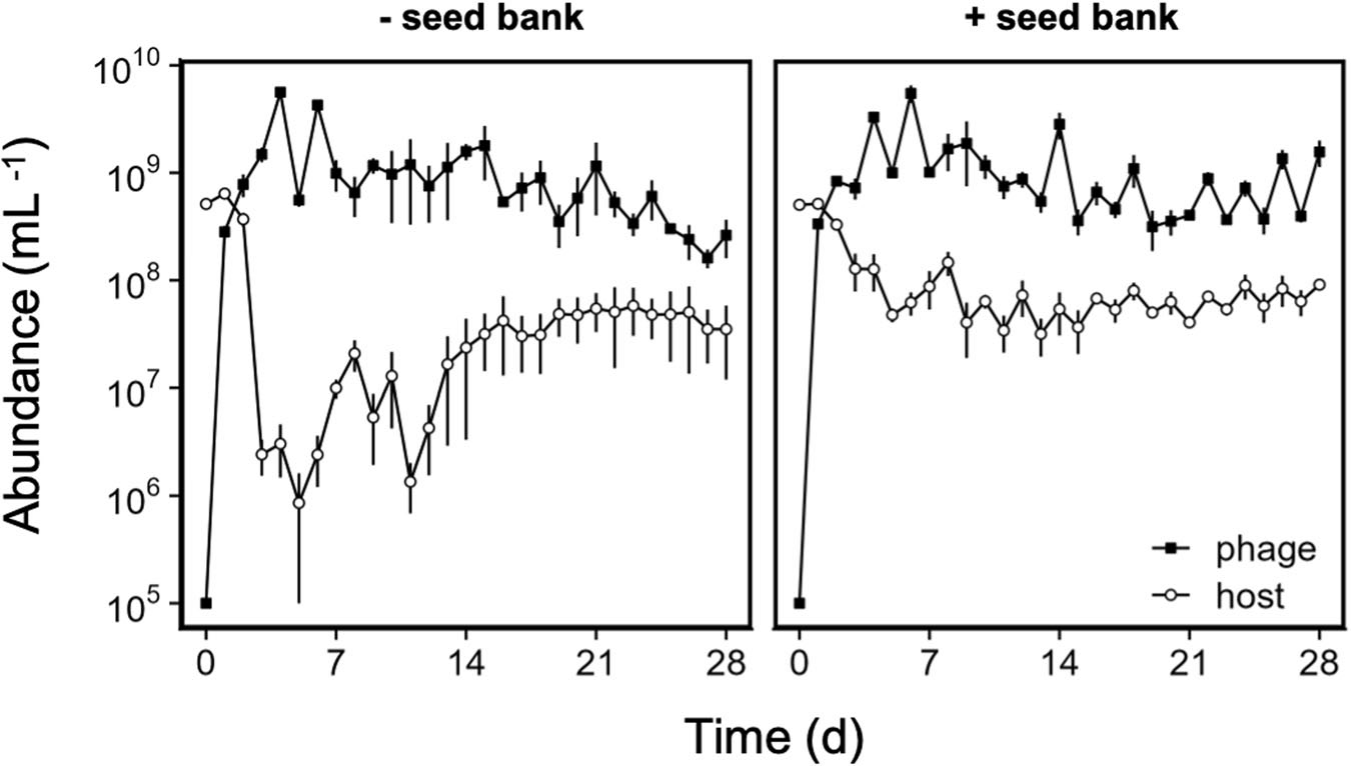
Seed banks altered host-phage population dynamics. Bacteria and phage dynamics were tracked in replicate (*n* = 3) populations that were propagated by serial transfer every two days (see Fig. 1). In the + *seed bank* treatment, the host could sporulate. In the - *seed bank* treatment, the host had an engineered mutation that prevented sporulation. Phage SPO1 was added to all populations (flasks) in the + *phage* treatment on day 0. See Fig. S5 to compare population dynamics of the different host strains in the - *phage* and + *phage* treatments. Data represented as mean ± SEM.

To test how the dormancy refuge affects antagonistic coevolution, we conducted a serial-transfer experiment where we challenged *B. subtilis* hosts with SPO1 phages in the presence or absence of an external seed bank (Fig. 1). The combination of strain genetics, growth medium, and transfer regime was effective in maintaining high sporulation levels over the course of the experiment. For example, in the + *seed bank* treatment, endospore abundances often exceeded the abundance of vegetative cells at the time of transfer (Fig. S3). The seed bank treatment altered how phage affected host dynamics (RM-ANOVA; phage x seed bank x time, *F*_28, 224_ = 2.2, *p* = 0.0009, Fig. 3). Without a seed bank, phage infection led to a 15-fold reduction in bacterial population size compared to non-infected control (*post hoc* comparisons based on estimated marginal means of the time series, *t*_8_ = 10.6, *p* < 0.0001) with minimum host densities (8.5 × 10^5^) occurring early in the experiment (day 5). With a seed bank, phage infection reduced average population sizes by only six-fold compared to non-infected controls (*post hoc* comparisons based on estimated marginal means of the time series, *t*_8_ = 9.7, *p* < 0.0001) with minimum host densities (3.2 × 10^7^) occurring later in the experiment (day 13). Seed banks also stabilized phage-induced fluctuations in host density (*post hoc* comparisons based on estimated marginal means, *t*_268_ = 3.0, *p* = 0.031). This effect was most pronounced early in the experiment, when phages had the largest influence on host population densities (Fig. S4). Midway through the experiment (day 14), phage-induced fluctuations in bacterial densities were dampened in both of the seed bank treatments. Without phage, seed banks had no effect on bacterial densities (*t*_8_ = 1.2, *p* = 0.28; Fig. S4), but they did increase population stability throughout the experiment (*t*_268_ = 12.5, *p* < 0.001, Fig. S4).

### Susceptible hosts persisted in seed banks

**Fig. 4:**
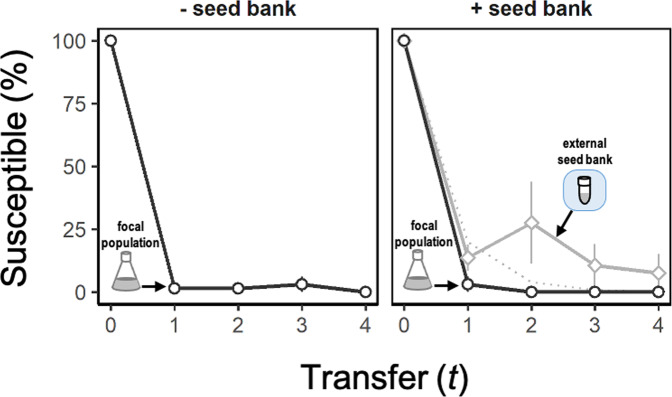
Retention of susceptible hosts with a seed bank. We quantified susceptibility by challenging clones of *Bacillus subtilis* against the ancestral phage. In both the + *seed bank* and - *seed bank* treatments, we performed this assay on clones (*n* = 22) that were isolated from each focal population contained in a flask (Fig. 1) just prior to serial transfer (). In addition, we revived clones (*n* = 22) from the external seed bank (Fig. 1) at each time point and challenged those against the ancestral phage (). The expected percentage of susceptible clones () is based on losses to dilution caused by serial transfer of clones originating from the pre-infection external seed bank (see Supplementary Information). Data represented as mean ± SEM of the replicate populations (*n* = 3).

To evaluate how seed banks affect the evolution of phage-resistance, we quantified host susceptibility to the ancestral phage for bacteria isolated from replicate populations over time. In the - *seed bank* treatment, phage susceptibility rapidly dropped to frequencies that were below detection. By the time of the first transfer, susceptible hosts were replaced by resistant bacteria that made up nearly 100% of the population and remained at this frequency for the remainder of the experiment (Fig. 4). A similar trend was observed in the + *seed bank* treatment for clones that were sampled from the total population (vegetative cells + endospores) prior to transfer (RM-ANOVA, *F*_1, 4_ = 1.1, *p* = 0.354). However, phage susceptibility in the external seed bank was significantly different from that of the total population (RM-ANOVA, *F*_1, 14_ = 12.5, *p* = 0.003). Specifically, more clones from the seed bank were susceptible than what would be expected given the dilution rate of endospores from the pre-infection seed bank (Fig. 4). This finding suggests that susceptible hosts were able to replicate and sporulate in the presence of viruses even when phage-resistance dominated the host population.

### Seed banks retained genetic diversity

**Fig. 5:**
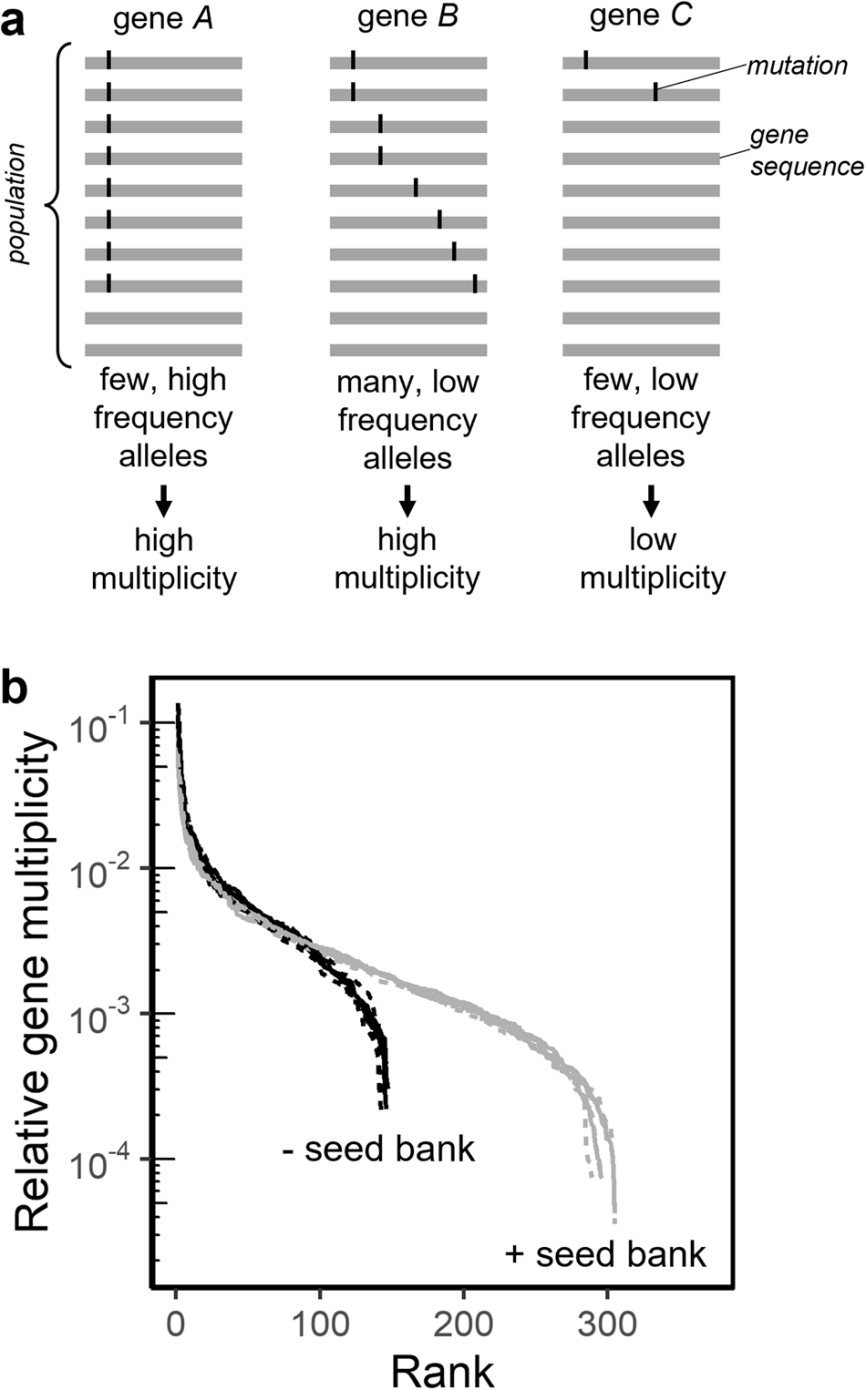
Rank-abundance distribution of mutations in host populations with and without a seed bank. **a** Mutations were identified by sequencing and mapped to the genes which they affect. The multiplicity of a gene reflects the number of mutations observed in a gene given its length and was weighed by the frequency of those mutations in the population. Given genes of equal length (genes *A*, *B* and *C*) high multiplicity can arise from high mutation frequency in the population (gene A), multiple mutated sites (gene B), or a combination of the two. **b** For comparison among populations, we calculated the relative multiplicity, by normalizing the sum of multiplicities in each population to equal one. Each curve represents the relative gene multiplicity ranked by decreasing multiplicity values for a single population. Solid lines represent populations from the + *phage* treatment (*n* = 3) while dashed lines represent populations from the – *phage* treatment (*n* = 3). The effect of seed banks on the distribution of multiplicity was determined using a permutational Kolmogorov-Smirnov test.

The distribution of host genetic variation (i.e., alleles) in bacterial populations was significantly altered by the seed bank treatment (Fig. 5). When examining gene multiplicity, which accounts for both the number of non-synonymous mutations per gene weighted by gene length and the frequency of mutations (Fig. 5a), populations evolving with a seed bank had roughly twice as many genes with mutations than those evolving without a seed bank. The additional genetic diversity from the seed bank resulted in a longer tail of multiplicity scores (Fig. 5b).

The effect of the seed bank on genetic diversity was also reflected in the composition of bacterial genes with mutations. More than 70% of the allelic variation among population could be attributed to the seed bank (Fig. S6. PERMANOVA *F*_1,8_ = 65.3, *p* < 0.0001). Seed banks retained allelic variants of genes that were involved in a wide range of functions (Table S3, Supplementary Text). For example, genes significantly correlated with the seed bank treatment in the ordination plot of host mutations were related to stress response (e.g., *fluC*, *yhdN*, *yceH*), cell wall synthesis (e.g., *dacA*, *ylmD*), and the regulation of gene expression (e.g., *yrdQ*). In contrast, the seed bank treatment had no effect on the distribution of allelic variants (Fig. S7) or on the composition of mutated genes (Fig. S8) in phage populations.

### Genetic targets of coevolution

**Fig. 6:**
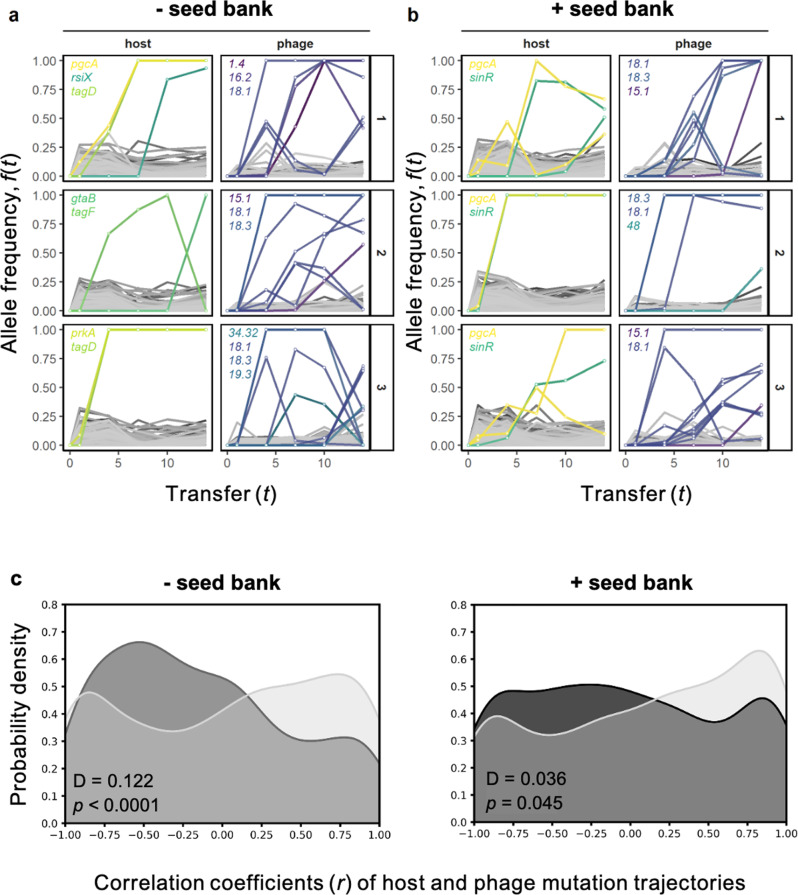
Seed banks dampened molecular coevolutionary dynamics between bacteria and phage. The frequency trajectories of mutant alleles in phage-infected communities (**a**) without a seed bank and (**b**) with a seed bank. Each row shows the data for the host and phage of a single community (numbers on right side). Non-synonymous mutations that reached a frequency >0.3 are colored by the gene in which it occurred. The names of genes with high-frequency mutations are provided for each population. See Table S4 for details on genes. **c** In the - *seed bank* treatment, the distribution of correlation coefficients between host and phage mutation trajectories skewed negative relative to a null distribution obtained by permuting time labels. In the + *seed bank* treatment, the distribution resembled that of the null with a slight overabundance of low correlation pairs, consistent with seed bank buffering of host-phage coevolutionary dynamics.

Phages also influenced the composition of host mutations (Figs. 6 and S6, PERMANOVA *F*_1,8_ = 6.1, *p* = 0.022). Nearly all mutations that reached high frequencies ( > 0.3) in phage-infected populations were in genes involved in teichoic acid biosynthesis. Teichoic acid is a polymer found in the cell wall of Gram-positive bacteria that is used by phage SPO1 for attachment [61]. All replicate populations in the + *phage* treatment had high frequency mutations in at least one of four genes (*pcgA*, *tagD, tagF* and *gtaB*) in the teichoic acid pathway (Figs. 6 and S9). Of these, *tagD* and *pcgA* mutations arose independently in separate populations, and were significantly correlated with phage infection in the ordination plot of host mutations (Table S3). Beside *pcgA* mutations, all populations with a seed bank that were infected by phage had high frequency mutations in the *sinR* repressor of biofilm formation. In the absence of phage, all populations had high frequency mutations in a single gene (*oppD*) of the *opp* oligopeptide transporter system, and four of six populations had high frequency mutations in the phosphorelay kinase *kinA* (Fig. S9). Without a seed bank, hosts had a greater number of high frequency mutations, including multiple genes of the *opp* operon and in *resE*, a sensor kinase regulating aerobic and anaerobic respiration. High frequency mutations in the phage populations were predominantly in genes encoding tail structural genes (*gp15.1, gp16.2, gp18.1, gp18.3*), irrespective of seed bank treatment (Fig. 6). Previous work with *B. subtilis* demonstrated that resistance to SPO1 caused by mutations in *gtaB* could be overcome by mutations in tail fiber genes *gp18.1, gp18.3*, as well as *gp16.2* [61].

### Seed banks affect coevolutionary dynamics

To determine how coevolution was affected by a seed bank, we quantified the correlation between trajectories of host and phage mutations over time [72]. If host and phage imposed reciprocal selection on each other, we would expect there to be a strong correlation between segregating mutations of the two populations. Without a seed bank there was an overabundance of negative correlations compared to a null distribution obtained via permutation (Fig. 6c). With a seed bank, the observed distribution of pairwise correlations was still significantly different from the null, but its form did not skew to one side, overall being more similar to the uniform shape of the null distribution (Fig. 6c). The distributions of pairwise correlations between host and phage mutation trajectories with and without a seed bank were significantly different from each other (Kolmogorov-Smirnov test, *D* = 0.151, *p* < 0.0001). Taken together, our analysis demonstrates that the correlations between phage and host mutational trajectories were weakened by the seed bank consistent with dormancy-dampened coevolutionary dynamics.

## Discussion

We experimentally tested how seed banks affect the coevolutionary dynamics between bacteria and phage populations by manipulating endosporulation, a dormancy trait that has important implications for the persistence and spread of bacteria in host-associated and environmental ecosystems. By altering adsorption rates and creating a seed bank, sporulation reduced bacterial mortality associated with phage infection. This in turn buffered population dynamics, preserved phage-susceptible phenotypes, and retained low-frequency mutations in the host populations. High frequency mutations in genes that are known to be targets of selection repeatedly arose in both host and phage populations. Through the analysis of mutational trajectories, our data suggest that seed banks provide physical protection and biological memory [17] that can dampen the strength of antagonistic coevolution between bacteria and phage populations.

### Sporulation provided a refuge against phage infection

Sporulation is a complex trait that allows bacteria to persist in fluctuating environments. We demonstrated that sporulation also provides a refuge from phage infection. Phage SPO1 was unable to attach to endospores, likely owing to modifications of the spore’s cell surface (Fig. 2). Endospores are encased in proteins that can mask the receptors that phages use to recognize and attach to the host cell. In addition, this spore coat protects the cell wall from lytic enzymes [28], such as those used by many phages for entry into the host cell [73]. Although transient, sporulation offers some advantages compared to other forms of phage defense. For example, it may allow the host to avoid costs associated with phage resistance mutations while also providing broad protection against multiple, or possibly all, phages. Protection is likely afforded by incompatible binding, but also the physical challenge associated with penetration of the thick spore coat [47, 74, 75]. We demonstrated defense against phage (Fig. 2), but protection may extend even further given that some predators (e.g., protists) are unable to digest endospores [28]. These often-overlooked features may help explain why spore-forming bacteria are one of the most abundant cell types on Earth [76]. However, the seed bank refuge is not restricted to endosporulation. Other forms of dormancy also provide resistance to pathogens and predators, including resting stages of bacteria, algae, plants, and metazoans [55, 77–80].

### Seed banks altered eco-evolutionary dynamics

Phage exert strong top-down pressure on bacteria that often results in complex eco-evolutionary dynamics [8, 81]. Within a single transfer post infection, phage resistance swept through host populations (Fig. 3) as is commonly observed in laboratory coevolution studies (e.g., 4). However, in the first transfer there was no single genotype that dominated the host populations in any of the infected populations (Fig. 6). Thus, the phenotypic response to the strong selection imposed by phages was achieved through genetic diversification that was only later purged when certain genotypes fixed in the host population. Regardless of its genetic basis, the rapid evolution of phage resistance allowed *Bacillus* to recover by the end of the first transfer, and achieve densities that were comparable to non-infected populations (Fig. S5). Despite the low frequency of sensitive hosts, phage population sizes remained high. One explanation for this pattern is that phage mutants evolved that could infect resistant bacteria. In support of this, we documented an increase in the frequency of mutations associated with host receptors (teichoic acids) as well as phage tail components that are involved in resistance-breaking [61]. Together, the mutations recovered in our study are associated with hallmark targets of coevolution [38, 44].

Seed banks significantly altered host-phage dynamics. Following the second transfer, the size of the infected host population was significantly reduced, an effect that persisted for the remainder of the experiment (Fig. 3). In the absence of a seed bank, phage infection led to larger and more rapid reductions in bacterial densities. This phage effect was much less pronounced in the presence of a seed bank, most likely due to lower per-capita mortality afforded by invulnerable endospores in the host population. In addition, the seed bank likely stabilized bacterial populations, which experienced fluctuating resource conditions during serial passage. Following each transfer, most endospores germinated. As unprotected and active cells, these individuals became more vulnerable to phage infection. Prior to transfer the following day, resource depletion would serve as a cue to initiate sporulation. In + *phage* treatment, this resulted in endospore densities that equaled or exceeded that of vegetative cells (Fig. S3). Such findings are consistent with predictions that a refuge in the form of invulnerable prey can reduce the amplitude predator-prey cycles [75, 82–84].

While sporulation is beneficial to *Bacillus* as a phage defense, our findings suggest, somewhat counterintuitively, that it may also promote the persistence of phage populations. In the seed bank, susceptible hosts attained higher frequencies for a longer duration of time (Fig. 4). Resuscitation of susceptible hosts should allow for more phage reproduction, offsetting losses due to washout and particle decay. Furthermore, in the absence of a seed bank, rare susceptible hosts are at higher risk of going locally extinct, especially when there is strong bottlenecking (e.g., serial transfer events) [85, 86]. As a consequence, seed banks may actually support the generation of phage diversity, which is critical for coevolution. Indeed, resistance-breaking mutants are more likely to emerge in a population containing both resistant and susceptible hosts [87, 88]. Overall, by stabilizing host populations and maintaining a subpopulation of sensitive hosts, seed banks may promote host-parasite coexistence, in part, through the rise of phage mutants that are required for coevolution.

### Seed banks altered the distribution of mutations

Consistent with expectations, our experiments revealed that seed banks maintain genetic diversity. The number of genes for which we detected allelic variants was roughly double in populations that had a seed bank, whether or not they were infected by phage (Fig. 5). Because our populations were initiated from single colonies, the observed host genetic diversity must have been generated de novo during the course of the experiment. The increased number of mutated genes in the + *seed bank* treatment was not simply a result of bacteria having a larger population size as evidenced by the fact that bacterial densities were similar in both phage treatments (Fig. S5). Similarly, the difference in mutated genes cannot be attributed to phage-accelerated diversification [89], since the seed bank effect on diversity was observed in infected and non-infected host populations. Rather, our results are consistent with a genetic storage effect, where rare alleles that would have otherwise been lost to genetic drift or negative selection were retained in the bacterial population. The effect on diversity recorded here at the population level is analogous to expectations of increased species richness and rarity in communities with a seed bank, which is suggested to explain long-tailed species abundance distributions observed in microbial systems [17, 90]. While the retention of genetic diversity is not due to phage infection dynamics, it has consequences for bacteria-phage coevolution. First, elevated host diversity can impede the spread of a parasite population [91, 92]. Second, prey diversity can create feedbacks that affect the dynamics and stability of predators and their prey [93]. Collectively, by providing a refuge from parasites, seed banks can stabilize the dynamics and increase the diversity of a host population, which has direct consequences for coevolution.

### Seed banks dampened coevolution

In a community of coevolving bacteria and phage, reciprocal selection should result in correlations between phage and host genotypes over time. Without a seed bank, we found that the relationship between derived alleles of host and phage resulted in a skewed distribution with strong negative correlations (Fig. 6c). With a seed bank, the correlation between segregating alleles in the phage and host populations was significantly weaker. Such decoupling likely reflects the dampening of phage selection on host variants due to the seed bank refuge. However, seed banks also have the potential to accelerate evolution by allowing rare variants from the past to resuscitate under conditions for which they are better adapted [94], which is a form of biological memory. For example, host resistance mutations can lurk at low frequencies before rising and altering the trajectory of bacteria-phage coevolution [95]. When these so-called “leapfrog dynamics” arise, coevolution proceeds by the occasional replacement of dominant host and parasite genotypes. In many laboratory-based evolution studies, reciprocal selection between hosts and phages gives rise to an arms race, which is accompanied by hard sweeps that lead to fixation [38, 39, 89]. Over time, these eco-evolutionary dynamics tend to be less pronounced as populations undergo diversification and experience trade-offs associated with resistance and counter resistance [46, 96, 97]. Seed banks provide another means of maintaining diversity, which may buffer bacteria-phage interactions based on our observation of stabilized dynamics (Fig. 3) and dampened correlations of mutational trajectories (Fig. 6). Future efforts to elucidate the net effect of seed banks on coevolution should combine phenotypic data of isolate-based infection networks with the genomic analysis of the host and parasite populations.

### Future directions and conclusions

External seed banks can be used to explore other ecological and evolutionary phenomena that emerge when a population has overlapping generations. Important features of a seed bank, including size and age structure, can be achieved by altering the sample volumes and mixing ratios of the external seed bank. While tractable for use with microorganisms, in principle, the approach is amenable for use with any group of taxa where individuals can be preserved in a suspended metabolic state, for example, through cryopreservation or lyophilization. Such strategies may allow for design of experiments to test theory that integrates complex life histories with demography and evolution (e.g., [94]). Because this approach had not previously been implemented, our experiment was designed to test fundamental expectations of seed bank theory in a controlled and replicated manner. As a consequence, many of the complexities of seed banks and coevolution that are common in environments like guts or soil were not explicitly captured in the current study. Nevertheless, the external seed bank bears similarity to naturally occurring seed banks where dormant individuals reside in patches that are spatially distinct from metabolically active individuals, such as plant seeds in soils or phytoplankton cysts in sediments [98–100].

Experiments like the ones described here could be expanded to explore other questions relating to the evolutionary ecology of seed banks. For example, seed banking is not limited to host populations. Parasites with dormant stages are also common, and in some systems, both hosts and parasites form seed banks. Furthermore, seed bank theory could be used for understanding reproduction-survival trade-offs associated with forms of viral dormancy such as lysogeny and latency. More work is needed to grapple with the complexity that can emerge under such conditions, but existing theory suggests that dormancy in host-parasite systems can feed back on the evolution of seed banking itself [34]. For example, in our study system, the seed bank refuge could contribute to the maintenance of endosporulation, a complex trait involving hundreds of genes. When spore-forming bacteria are maintained for many generations in favorable environments, random mutations ultimately hit essential sporulation genes leading to the loss of this trait [53, 101]. Last, there is growing evidence that dormancy may interact with dispersal by facilitating the movement and colonization of organisms in spatially variable landscapes [102]. Experiments like the ones described here would provide a means of testing such ideas, which would be important for understanding epidemics and disease dynamics in more complex settings [17].

In summary, dormancy is a life-history strategy that is widely distributed throughout the tree of life. It can lead to the generation of a seed bank, which generates structure and memory to a population. As a result, seed banks modify demography and diversity in ways that buffer populations against unfavorable and fluctuating environmental conditions. Seed banks also alter interactions among individuals belonging to different species, which has implications for mutualistic and antagonistic dynamics. Our study demonstrated that seed banks can create a refuge that stabilizes host populations when challenged by phages. Protection provided by dormancy can retain genetic and phenotypic diversity of host populations with implications for eco-evolutionary feedback. There is evidence, however, that parasites can exploit bacterial dormancy in ways that enhance reproductive or survivorship components of fitness [31, 103–105]. For example, phages can acquire sporulation genes, which suggests that dormancy may play an important role in bacteria-phage coevolution [56, 57]. Similar lines of investigations in other study systems will help reveal the extent to which seed banks influence the coevolutionary process.

## Supplementary information

Supplementary Information

## Data Availability

Sequence data are available on NCBI SRA (BioProject PRJNA932315). Code and data to reproduce all analyses are available on Zenodo (10.5281/zenodo.7786196) as well as on GitHub (https://github.com/LennonLab) in the following repositories: *coevolution-ts*, *coevo-seedbank-seq*, *coevo-seedbank-ancestors*, and *Phage_spore_adsorption*.
